# Supplementary material for: Identification of photoperiod-induced specific miRNAs in the adrenal glands of Sunite sheep (Ovis aries)
Source: Front Vet Sci. 2022 Jul 22;9:888207. doi: 10.3389/fvets.2022.888207 (PMC9354845; doi:10.3389/fvets.2022.888207)
Supplement: Supplementary file 1 [file Data_Sheet_1.ZIP › Supplementary file/miRDeep2 file.zip!/miRDeep2 file/Know_Structure/SPLP42A1/oar-let-7b.pdf]

miRBase precursor : oar-let-7b
Total read count : 189864
oar-let-7b read count : 189282
remaining reads : 582

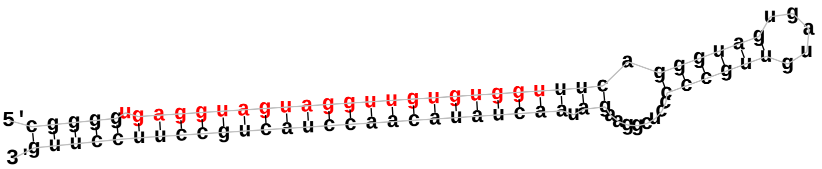

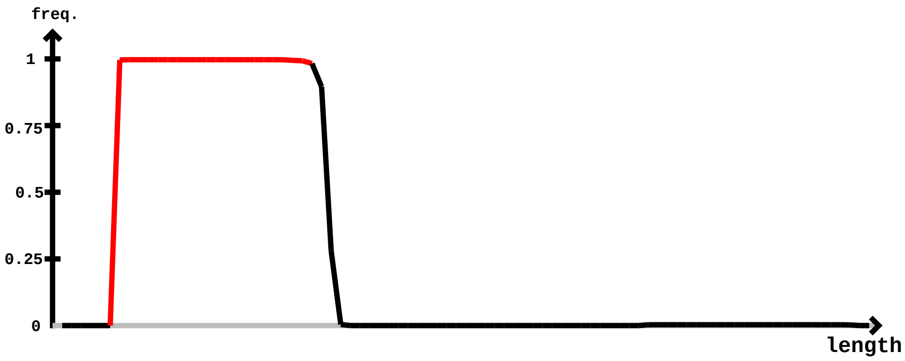

oar-let-7b

```
5'- cggggugagguaguagguugugugguuucaggguagugauguugcccccucggaagauaacuauacaaccuacugccuuccuug   -3'    exp
    (((((.(((((((((((((((((((((((.((((((.....))))))(....)..)).))))))))))))))))))))))))))    reads      mm         sample
    ....gugagguaguagguugu...............................................................    1          0          seq
    ....gugagguaguagguugugugguu.........................................................    1          0          seq
    .....ugaCguaguagguugug..............................................................    1          1          seq
    .....ugagguaguagguugug..............................................................    48         0          seq
    .....ugagguaguagguugugu.............................................................    438        0          seq
    .....ugagguaguagguugugug............................................................    284        0          seq
    .....ugagguaguagguugugugg...........................................................    1927       0          seq
    .....ugagguaguagguugugugguu..........................................................    16489      0          seq
    .....ugagguaguagguugugugguu.........................................................    116841     0          seq
    .....ugagguaguagguugugugguuu........................................................    52352      0          seq
    .....ugagguaguagguugugugguuuc.......................................................    758        0          seq
    .....ugagguaguagguugugugguuuca......................................................    2          0          seq
    ......gagguaguagguugugu.............................................................    1          0          seq
    ......gagguaguagguugugugg...........................................................    2          0          seq
    ......gagguaguagguuguguggu..........................................................    10         0          seq
    ......gagguaguagguugugugguu.........................................................    75         0          seq
    ......gagguaguagguugugugguuu........................................................    46         0          seq
    .......agguaguagguugugugguu.........................................................    2          0          seq
    .........guaguagguugugugguu.........................................................    1          0          seq
    ..........uaguagguugugugguu.........................................................    1          0          seq
    ..........uaguagguugugugguuA........................................................    1          1          seq
    ...........aguagguugugugguuu........................................................    1          0          seq
    ........................................guugcccccucggaagauaacu......................    1          0          seq
    .............................................ccccucggaagauaacuauaca.................    1          0          seq
    ......................................................agauaacuauacaaccuacugccu......    1          0          seq
    ..........................................................aacuauacaaccuacugccuuc....    1          0          seq
    ...........................................................acuauacaaccuacugccuucc...    1          0          seq
    ............................................................cuauacaaccuacugccuu.....    1          0          seq
    ............................................................cuauacaaccuacugccuuc....    9          0          seq
    ............................................................cuauacaaccuacugccuucc...    98         0          seq
    ............................................................cuauacaaccuacugccuuccu..    444        0          seq
    ............................................................cuauacaaccuacugccuuccuu.    19         0          seq
    .............................................................uauacaaccuacugccuucc...    1          0          seq
    .............................................................uauacaaccuacugccuuccu..    5          0          seq
```
